# Transcriptional Auto-Regulation of RUNX1 P1 Promoter

**DOI:** 10.1371/journal.pone.0149119

**Published:** 2016-02-22

**Authors:** Milka Martinez, Marcela Hinojosa, Daniel Trombly, Violeta Morin, Janet Stein, Gary Stein, Amjad Javed, Soraya E. Gutierrez

**Affiliations:** 1 Departamento de Bioquimica y Biologia Molecular, Facultad de Ciencias Biologicas, Universidad de Concepcion, Concepcion, Chile; 2 Department of Cell and Developmental Biology, University of Massachusetts Medical School, 55 Lake Avenue North, Worcester, 01655, Massachusetts, United States of America; 3 Department of Biochemistry and Vermont Cancer Center, University of Vermont College of Medicine, 89 Beaumont Avenue, Burlington 05405, Vermont, United States of America; 4 Department of Oral and Maxillofacial Surgery, School of Dentistry, University of Alabama at Birmingham, Alabama, United States of America; University of Massachusetts Medical, UNITED STATES

## Abstract

RUNX1 a member of the family of runt related transcription factors (RUNX), is essential for hematopoiesis. The expression of RUNX1 gene is controlled by two promoters; the distal P1 promoter and the proximal P2 promoter. Several isoforms of RUNX1 mRNA are generated through the use of both promoters and alternative splicing. These isoforms not only differs in their temporal expression pattern but also exhibit differences in tissue specificity. The RUNX1 isoforms derived from P2 are expressed in a variety of tissues, but expression of P1-derived isoform is restricted to cells of hematopoietic lineage. However, the control of hematopoietic-cell specific expression is poorly understood. Here we report regulation of P1-derived RUNX1 mRNA by RUNX1 protein. *In silico* analysis of P1 promoter revealed presence of two evolutionary conserved RUNX motifs, 0.6kb upstream of the transcription start site, and three RUNX motifs within 170bp of the 5’UTR. Transcriptional contribution of these RUNX motifs was studied in myeloid and T-cells. RUNX1 genomic fragment containing all sites show very low basal activity in both cell types. Mutation or deletion of RUNX motifs in the UTR enhances basal activity of the RUNX1 promoter. Chromatin immunoprecipitation revealed that RUNX1 protein is recruited to these sites. Overexpression of RUNX1 in non-hematopoietic cells results in a dose dependent activation of the RUNX1 P1 promoter. We also demonstrate that RUNX1 protein regulates transcription of endogenous RUNX1 mRNA in T-cell. Finally we show that SCL transcription factor is recruited to regions containing RUNX motifs in the promoter and the UTR and regulates activity of the RUNX1 P1 promoter *in vitro*. Thus, multiple lines of evidence show that RUNX1 protein regulates its own gene transcription.

## Introduction

Runt-related transcription factor 1 (RUNX1) belongs to a family of three transcription factors. All three members share homology in a 128 amino acids region designated as runt homology domain (RHD), which mediates the binding to consensus core sequence 5’-PuACCPuCA-3’ in the target DNA. RHD is also required for nuclear import, interaction with core binding factor β (CBFβ) for an efficient binding to target DNA, and physical and functional interaction with several other proteins to regulate gene transcription [1, 2]. Members of RUNX family are key regulators of lineage-specific gene expression and development of distinct organs [2, 3]: RUNX1 is essential for definitive hematopoiesis during embryonic development [4–6], RUNX2 is required for osteogenesis [7–9] and RUNX3 for development of gut and proprioceptive neurons of the dorsal root ganglia [10–13]. Thus, despite the presence of evolutionary conserved RHD, RUNX family members exhibit distinct and non-redundant biological functions.

Global deletion of RUNX1 gene results in embryonic lethality at midgestation due to hemorrhages in the central nervous system [4, 5]. In adult mice, RUNX1 is required for development and maturation of thymocytes, T and B lymphocytes, as well as megakaryocytes [14–16]. Conditional deletion of RUNX1 gene in hematopoietic organs revealed that in early postnatal life RUNX1 is not essential for maturation of myeloid lineage cells or the maintenance of hematopoietic stem cells [14]. In contrast, in adult animals hematopoietic tissue specific loss of RUNX1 results in progressive splenomegaly, expansion of the myeloid compartment, cytopenia in the peripheral blood and increased fraction of the immature cells in the bone marrow [16]. Thus, RUNX1 continue to play an important regulatory function in adult hematopoiesis and postnatal development.

In leukemia RUNX1 gene is one of the most frequent targets of mutations and chromosomal rearrangements. In human, rearrangements of RUNX1 locus are associated with 30% of all acute leukemia [17–19]. Indeed, RUNX1 gene is involved in multiple leukemia associated chromosomal translocations (8;21) RUNX1-ETO, (16;21) RUNX1-MTG16, (3;21) RUNX1-Evi1, (12;21) TEL-RUNX1, and (X;21) RUNX1-FOG2 [20, 21]. The resultant fusion proteins are involved in leukemiogenesis with a wide range of pathological features. For example, t(8;21) RUNX1-ETO tends to occur in early adulthood and is characterized by enhanced granulopoiesis and inhibition of erythropoiesis. RUNX1-ETO is found in 12–15% of patients with *de novo* acute myeloid leukemia [22].

Dysregulation of RUNX1 gene also results in development of other hematological disorders such as Myelo Dysplastic Syndrome (MDS), Acute Lymphoblastic Leukemia (ALL) and Familial Platelet Disorder (FPD). Somatic mutations in the RUNX1 gene is one of the major driving factors in the etiology of the MDS which is characterized by 20% blasts in the blood or bone marrow. FPD is characterized by haploid insufficiency mutation of RUNX1 gene with qualitative and quantitative defects in platelet. FPD patients show high frequency (20–50%) of acute myeloid leukemia development [23–25]. Thus, dominant inhibition of RUNX1 function is considered a common, and necessary, alteration for the development of several hematological disorders.

The RUNX1 gene locus spans 260kb on human chromosome 21. RUNX1 expression is regulated by a proximal P2 and distal P1 promoter [26]. The P1 promoter resides 160kb upstream of the P2 promoter. Multiple RUNX1 mRNA species are derived from alternative splicing and differential utilization of the two promoters [26]. The P2 promoter-derived isoforms are principally expressed in non-hematopoietic tissues such as brain, kidney, pancreas, heart and liver [27]. The isoform expressed from the P1 promoter, encode a 480 aa RUNX1 protein, while the isoform expressed from P2 promoter lack the first exon and encode a 453 aa protein. The P1 RUNX1 isoform is predominantly expressed in hematopoietic stem cell, megakaryocytes and T lymphocytes present in thymus and spleen, [27–30]. Despite the importance of RUNX1 transcription factor in hematopoiesis, the regulatory mechanism and the factors involved in controlling *RUNX1* gene transcription remains poorly understood.

In addition to RUNX1, several nuclear regulators such as GATA1, PU.1 and SCL play important roles in the RUNX1 gene transcription during hematopoiesis. For instance, the SCL, a basic helix loop helix containing transcription factor, is predominantly expressed in hematopoietic tissues [31]. SCL also interacts with RUNX1 protein to form regulatory complexes for gene transcription. Gene knockout studies have stablished critical requirement of SCL in regulating hematopoiesis specific gene expression and establishment of the vascular system [32–35]. SCL null embryos show a complete absence of blood formation and display defects in yolk sac angiogenesis [35, 36]. SCL exerts its activity in hematopoietic progenitors as part of a multiprotein complex. In erythroid cells SCL interacts with other transcription factors such as I47, Limb domain binding protein (LDB1), limb only domain protein (LMO 2), GATA1 and RUNX1 [37–39].

In this paper we have investigated the role of RUNX1 protein in transcriptional regulation of P1 promoter. Through the use of chromatin immunoprecipitation assays, we show in hematopoietic cells that RUNX1 protein is bound to multiple sites situated in P1 promoter and first exon. Site directed mutagenesis revealed that the promoter and UTR RUNX regulatory motifs show a differential response. RUNX motifs in the promoter enhance transcription, whereas RUNX motifs in the UTR inhibit gene transcription. In hematopoietic cells a positive feedback loop regulates RUNX1 gene transcription.

## Methods

### Cell culture

The human myeloid HL-60 cells and Jurkat T-cells were obtained from The European Collection of Cell Cultures (ECACC), cultured in RPMI media supplemented with 10% fetal bovine serum (FBS), 2 mM L-glutamine, 100 U/mL penicillin, and 100 mg/mL streptomycin. Human cervical carcinoma, HeLa cells, which do not express RUNX1, were obtained from Stein’s lab, grown in DMEM supplemented with 10% FBS, 2 mM L-glutamine, 100 U/mL penicillin, and 100mg/mL streptomycin. Cells were maintained at 37°C in a humidified atmosphere with 95% air and 5% CO_2_.

### Cloning of the RUNX1 P1 promoter and expression vectors

Human RUNX-P1 promoter was amplified using genomic DNA isolated from HL-60 cells. The ∼1.1kb genomic fragment spans 591bp of P1 promoter, 444bp of the UTR and 41bp of coding sequence. The P1 promoter and UTR fragment (P1+UTR) was amplified using forward and reverse primers indicated in Table 1. In addition, the -591 to +54bp of RUNX1 P1 promoter was amplified using forward and reverse primers described in Table 1.

**Table 1:** List of Primers.

**Promoter Cloning**		
P1	Forward	5’GTTGTCCATTTAGGGGGAATAAAA3’
	Reverse	5’GGGTACGAAGGAAATGACTCAAATA3’
P1+UTR	P1 Forward	5’GTTGTCCATTTAGGGGGAATAAAA3’
	UTR Reverse	5’GCCCAAAGAAGTTTTCACACAA3’
**Cloning of RUNX Mutant Promoters**		
Site 3 Product 1 (667 bp)	Forward	5’GTTGTCCATTTAGGGGGAATAAAA3’
	Reverse	5’TGGTTCTGTGtgTGTTTATGAGG3’
Site 3 Product 2 (449 bp)	Forward	5’CTCATAAACAcaCACAGAACCAC3’
	Reverse	5’GCCCAAAGAAGTTTTCACACAA3’
Site 3, 4 Product 1 (764 bp)	Forward	5’CTAGCAAAATAGGCTGTCCC3’
	Reverse	5’TACCCAACTTGtgTGTCTGTGtgTGTTTATGAGGCCC3’
Site 3, 4 Product 2 (535 bp)	Forward	5’GGCCTCATAAACAcaCACAGACAcaCAAGTTGGGTAG3’
	Reverse	5’GCCCAAAGAAGTTTTCACACAA3’
Site 5 Product 1 (859 bp)	Forward	5’GTTGTCCATTTAGGGGGAATAAAA3’
	Reverse	5’AAACCCTGTGtgTTGCATTCAG3’
Site 5 Product 2 (357 bp)	Forward	5’CTGAATGCAAcaCACAGGGTTT3’
	Reverse	5’GCCCAAAGAAGTTTTCACACAA3’
**Primers for real time PCR**		
RUNX Site 1	Forward	5’GTTGTCCATTTAGGGGGAATAA3’
	Reverse	5’TTGGTAACGTCTATCATGGCATA3’
RUNX Site 2	Forward	5’AATCAGTAGTTCCAAAAACCACAA3’
	Reverse	5’CAGGCTGTGCAAGAAAATAGC3’
RUNX Site 3,4,5	Forward	5’GAAAACTTCTTTGGGCCTCAT3’
	Reverse	5’CTGTGGGTTGGTGATGCTC3’
SCL Site	Forward	5’TTTTCTTGCACAGCCTGGGGGAG3’
	Reverse	5’GCCCAAAGAAGTTTTCACACAACCC3’
SCL Site +23 enhancer	Forward	5’AACTGCCGGTTTATTTTTCG3’
	Reverse	5’TCTCTGGGAAGCCTCTTGAC3’
Control 1	Forward	5’TACCTGTGAGTTGCCAGCCCGT3’
	Reverse	5’GGCTACCCAACTTGTGGTTC3’
Control 2	Forward	5’TACCTGTGAGTTGCCAGCCCGT3’
	Reverse	5’CAGGCTGTGCAAGAAAATAGC3’
RUNX1 hnRNA	Forward	5’CGATGGCTTCAGACAGCATA3’
	Reverse	5’GGTGAAACAAGCTGCCATTT3’

The pGL3 Luciferase reporter plasmid was digested with SmaI to produce a blunt end vector. The 1.1kb and 645bp blunt end PCR products were ligated into the pGL3 Luc vector. The integrity of the sequences and correct orientation of the P1+UTR-Luc and P1-Luc plasmids was confirmed by direct sequencing. Site directed mutagenesis was utilized to introduce mutation in the three RUNX motifs located in the UTR. To introduce the mutation in RUNX site 3, complementary oligonucleotides carrying the two base pair mutation were synthesized. Two independent PCR fragments containing overlapping sequences of mutated RUNX site 3 were generated by using complementary oligos and the forward and reverse primers used for P1+UTR promoter amplification. The two PCR products of 661bp and 434bp were gel purified, combined and hybridized to generate P1+UTR template. The 1.1kb P1+UTR fragment carrying the mutation in RUNX site 3 was then amplified using the same forward and reverse primer as described above. To generate RUNX site 3,4 double mutant complementary oligonucleotides carrying mutations in both RUNX sites (see Table 1) were synthesized. A similar two step strategy described for RUNX site 3 mutant was used to generate 1.1kb P1+UTR fragment carrying mutation in both RUNX sites 3 and 4. The single and double mutant PCR products were ligated into SmaI digested pGL3 Luc plasmid. To generate RUNX site 3–5 triple mutant, a complementary oligonucleotide carrying two base pair mutation in RUNX site 5 (see Table 1) was synthesized and the same two step strategy was employed using site 3,4 mutant plasmid as template DNA. Incorporation of the substitution mutations in RUNX sites was confirmed by direct sequencing. The CMV-driven RUNX1 expression construct was described previously [40]. The expression vector carrying full length SCL cDNA was purchased from Origene (Rockville, MD, USA).

### Transient transfections and reporter assays

HeLa cells cultured in 12-well plates were transiently transfected with NanoJuice^™^ Transfection reagent (EMD Millipore, Pillerica, MA, USA), as per manufacturer’s instructions. Briefly, the cells were co-transfected with 200ng of promoter reporter, and increasing concentrations of RUNX1 and SCL expression vectors and a fix amount (5ng) of *Renilla* luciferase plasmid (Promega Corp. Madison, WI, USA) used as internal control. The transfected DNA was maintained at a constant total amount of 1400ng by using pBluescript plasmid (Agilent Technologies Inc, Santa Clara, CA, USA) as a filler DNA.

Jurkat and HL-60 cells were electroporated with P1+UTR-Luc and P1-Luc to determine basal activity of RUNX1 promoter. Briefly, 1×10^6^ cells were re-suspended in 100μl of Neon transfection buffer and electroporated with 10μg of each promoter reporter plasmid and 50ng of *Renilla* luciferase plasmid. Electroporated cells were seeded in 6-well plates and harvested 18 hours later. Cell pellets were collected by centrifugation and lysed and suspended in 50μL of passive lysis buffer (Promega Corp. Madison, WI, USA). Luciferase activity was determined in 20μl of cell lysates using the Dual-Luciferase Reporter Assay System (Promega Corp. Madison, WI, USA). Promoter-luciferase activity was normalized with *Renilla* luciferase values obtained in each sample.

### Chromatin immunoprecipitation

Chromatin immunoprecipitation (ChIP) assays were performed as described earlier [41] with some modifications. Jurkat cells (10×10^6^) were fixed with 1% formaldehyde at 37°C for 10 min. Crosslinking was stopped by using a final concentration of 0.125M glycine and cells were collected by centrifugation. Cells were washed with ice cold PBS, and re-suspended in 1mL of lysis buffer (25mM HEPES pH7.8, 1.5mM MgCl2, 10mM KCl, 0.1% (v/v) NP-40, 1X Cømplete, 25mM MG132) on ice for 10 min. Following dounce homogenization (20 strokes, pestle A), nuclei were collected by centrifugation at 750g for 5 min. Nuclear pellets were re-suspended in 1mL sonication buffer (50mM HEPES pH7.9, 140mM NaCl, 1mM EDTA, 1%(v/v) Triton X-100, 0.1%(v/v) Na-deoxycholate, 0.1%(v/v) SDS, 1X Cømplete, 25mM MG132) and sonicated on ice to an average DNA size of 200-300bp. The samples were centrifuged at 16,000 g for 15 min and precleared with A/G plus- agarose beads precoated with 2μg/mL sonicated salmon sperm DNA, and 1mg/mL BSA. For immunoprecipitation 3 units (A260) of pre-cleared chromatin was mixed with 2μg of RUNX1 polyclonal antibody (Active Motif, Carlsbad, CA), for 12 hours at 4°C. The immuno complexes were collected by binding to A/G plus- agarose beads for 1h at 4°C. The beads were washed twice with each of the following buffers: sonication buffer, sonication buffer containing 500mM NaCl, LiCl buffer (20mM Tris pH 8, 1mM EDTA, 250mM LiCl, 0.5% NP-40, 0.5% Na-deoxycholate) and 10mM Tris pH 8. The immunocomplexes were eluted in 50mM Tris pH 8, 1mM EDTA and 1% SDS at 65°C for 15 min. To reverse the crosslinking eluted chromatin was incubated with 200mM NaCl at 65°C for 12 hours. The immunoprecipitated DNA was purified by ethanol precipitation and 2μl of DNA was used for PCR reactions. Occupancy of RUNX1 protein was determined on promoter and UTR by independent PCR reactions. The forward and reverse primers for site 1, site 2 and sites 3,4,5 are described in Table 1. Primer sequences used to determine SCL occupancy in the RUNX1-P1 promoter and +23 enhancer located in intron 1 of the RUNX1 gene are described in Table 1. Data was analyzed with Prism 6 software, and statistical significance determined by student t-test.

### RNA interference, antibodies and western blot analysis

Jurkat cells were electroporated with 80nM of siRNA against human RUNX1 using the Amaxa Nucleofector system (Lonza Inc., Allendale, NJ, USA). Multiple siRNA targeting RUNX1 mRNA (Dharmacon Inc, Lafayette, CO) were initially screened. Only the siRNA with greater than 50% knock down efficiency of RUNX1 protein, were used for subsequent experiment. Briefly, 1×10^6^ Jurkat cells were electroporated with either a control or two different RUNX1 siRNA (siR1, siR2) for 18 hours. Cells were harvested for either ChIP or western blot analysis.

Blots were probed with polyclonal RUNX1 antibody (Cell Signaling Technology Inc, Beverly, MA) or SCL antibody (Santa Cruz Biotechnologies, Santa Cruz, CA). Blots were stripped and re-probed with polyclonal Actin, Lamin B antibody or mouse monoclonal GAPDH antibody (Santa Cruz Biotechnologies, Santa Cruz, CA). Protein were detected using species matched HPR-conjugated secondary antibodies and chemiluminiscence imagining.

### Real time reverse transcription—PCR

Jurkat cells were electroporated with siRNA as described above and RNA was extracted using RNeasy^®^Mini Kit (Qiagen Inc., Valencia, CA) according to the manufacturer’s protocol. The purified DNA-free hnRNA were used for synthesis of cDNA using SuperScript^™^ First-Strand Synthesis System (Life Technologies, Grand Island, NY) as per manufacturer’s instructions.

The relative level of P1 derived RUNX1 hnRNA was determined with a forward primer located in exon 1 and a reverse primer located in intron 1 (see Table 1). RT-PCR for RUNX1 hnRNA was used to detect transcriptional activity as a substitute for the nuclear run-on assay. The level of RUNX1-hnRNA was determined using ΔΔ Ct method with relative quantification to GAPDH used as an internal control.

## Results

### RUNX motifs regulate activity of the RUNX1 P1 promoter

**Fig 1:**
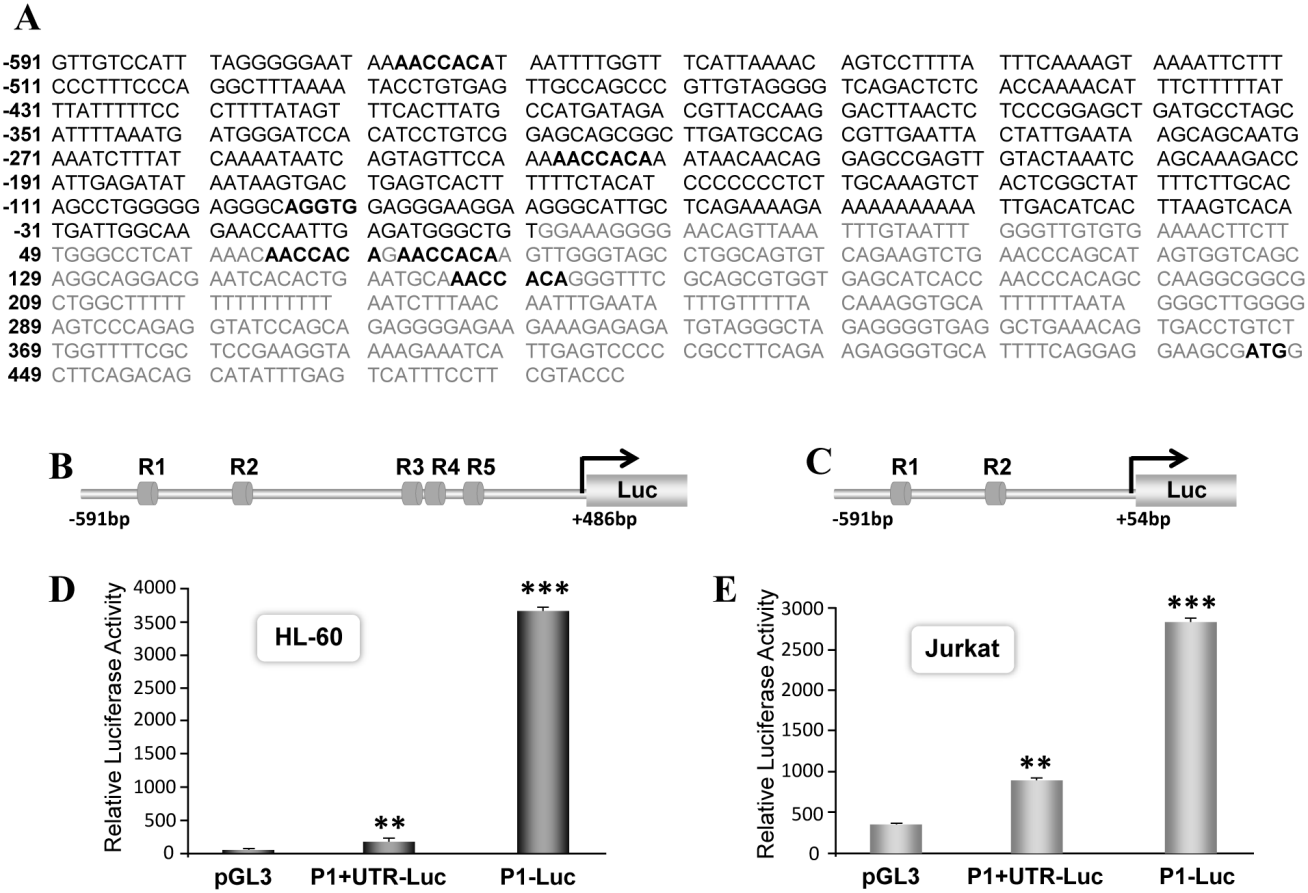
RUNX motifs regulate basal activity of the RUNX1 P1 promoter. A) The sequences of 591bp P1 promoter and 445bp UTR along with 41bp of coding region of the RUNX1 gene are shown. Binding sites for RUNX and SCL transcription factors are indicated in bold letters. The 5’UTR sequences are represented in gray color and translational start site ATG is indicated in bold letters. Diagrammatic representation of the RUNX1 P1 promoter (B) with UTR sequences and (C) without the UTR sequences. Relative position of five RUNX binding motifs labeled as R1-R5 is indicated with dark gray boxes. The basal activity of RUNX1 P1+UTR and RUNX1 P1 promoter in hematopoietic (D) HL-60 and (E) Jurkat cells is shown. D) HL-60 cells were electroporated with 200ng of either P1+UTR-Luc or P1-Luc promoter plasmids. Parallel plate of cells were transfected with promoterless pGL3-Luc plasmid as a baseline control. Cells were harvested 18h later to determine luciferase activity. Data were normalized with Renilla luciferase values used as an internal control. The normalized data from four independent experiments performed in triplicate is pooled and presented in bar graph with standard error of the mean. E) Jurkat cells were electroporated with 200ng of either P1+UTR-Luc or P1-Luc plasmids. Cells were harvested 18h later and promoter reporter activity determined as described above. Asterisks indicate statistically significant increase in basal promoter activity in HL-60 and Jurkat cells (**p<0.01, ***p<0.001).

**Fig 2:**
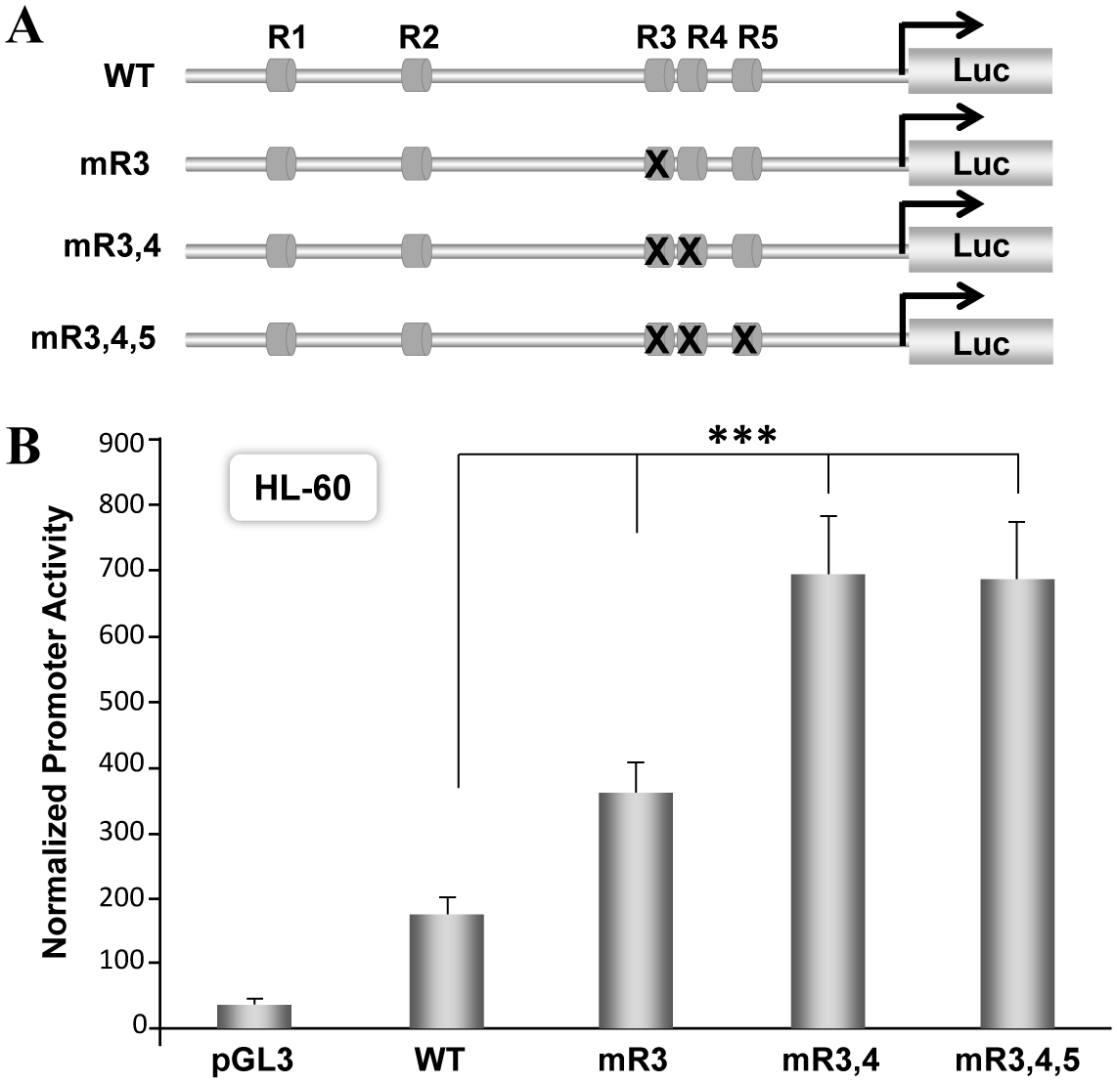
RUNX binding sites within 5’UTR inhibit RUNX1 P1 promoter activity. A) Schematic illustration of the wild type and RUNX sites mutated promoters. Mutant promoters were generated by introducing two base pair substitution mutations in one (mR3), two (mR3,4) or three RUNX sites (mR3,4,5) in the UTR region. Bases selected for mutation in each RUNX binding site are indicated in lower case (AACCACA→AcaCACA). B) HL-60 cells were electroporated with 200ng of either wild type or mutated promoter reporters. Cells were harvested 18h later and equal amount of cell lysates were evaluated for luciferase activity using the dual luciferase reporter system. Normalized values from four independent experiments with three replicates each are presented in bar graphs with standard error of the mean. Asterisks represent statistically significant increase in basal activity of the RUNX1 mutant promoters compared to wild type promoter (***p<0.001).

To identify potential transcription factors that regulates expression of RUNX1 mRNA derived from P1 promoter we performed *in silico* analysis. Genomic sequences of the human RUNX1-P1 promoter were screened for putative motifs of DNA binding proteins using TFSEARCH program. Multiple binding sites for hematopoietic transcription factor GATA1 and RUNX1 were identified in both the promoter and the proximal part of the untranslated region (Fig 1A). Two RUNX binding sites (R1 and R2) are located within 570bp of the P1 promoter (Fig 1C). Evolutionary conservance of these RUNX motifs among mammalian species suggest a possible autoregulatory function for the RUNX1 transcription factor. Since RUNX motifs were located both up and downstream of the transcription start site, we cloned RUNX1-P1 promoter with and without 486bp of exon 1 sequences (Fig 1B and 1C). We initially assessed basal activity of these promoters in hematopoietic HL-60 and Jurkat cells that endogenously express RUNX1 gene and have no abnormalities in the RUNX1 locus [42]. The 1.1kb fragment containing RUNX1 P1 promoter and UTR showed 4.6-fold increased luciferase activity compared to a promoterless pGL3 reporter vector (Fig 1D). Interestingly, removal of 432bp of the UTR resulted in a 20-fold increase in P1 promoter activity. We find similar pattern of RUNX1 promoter response in Jurkat cells (Fig 1E). To understand if RUNX motifs in the 5’UTR are contributing to transcriptional response, we performed site directed mutagenesis. RUNX1 P1+5’UTR promoters were generated carrying mutations in either single, double or all three RUNX motifs (Fig 2A). Activities of these mutated promoters were assessed in HL-60 and Jurkat cells. Mutation of first RUNX motif in the UTR (R3) resulted in 2-fold increase in basal activity when compared to the wild type P1+UTR promoter. Mutation of first and second sites (R3, R4) or all three RUNX motifs (R3,R4,R5) showed a 3.8-fold increase in promoter activity (Fig 2B). Similar increase in activities of mutant RUNX promoters were noted in Jurkat cells (data not shown). These results strongly suggest that RUNX motifs in the UTR inhibit activity of the RUNX1 P1 promoter.

**Fig 3:**
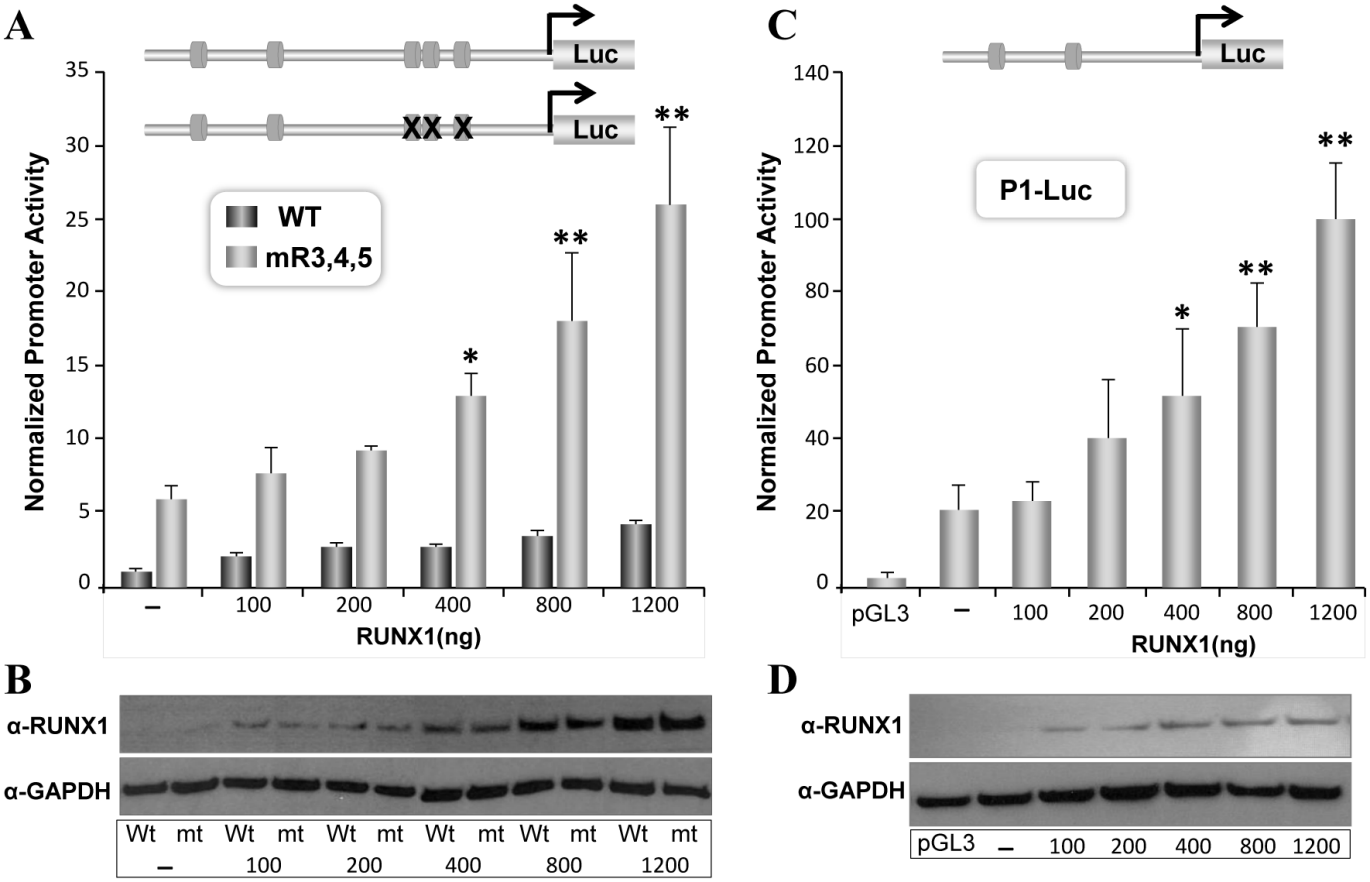
Mutation or deletion of RUNX motifs in the UTR enhances RUNX1 mediated transcriptional activity of the P1 promoter. A) Non-hematopoietic HeLa cells were transiently co-transfected with a fix amount (200ng) of either wild type or RUNX mutant promoter-reporter plasmids and increasing concentration (100-1200ng) of RUNX1 expression vector. Cells were harvested 18h later and luciferase activity was determined by dual luciferase assay system. Values were normalized with Renilla luciferase activity used as an internal control. Pooled values from at least four independent experiments performed in triplicate are presented in the bar graph along with the standard error of the mean. Asterisks indicate statistical significance between wild type and mutant promoter (*p<0.1, **p<0.01. B) Parallel plates of HeLa cells were transiently co-transfected exactly as described in (A) and harvested 18h later. Cell were directly lysed and equal amount of proteins were resolved by SDS-PAGE. Blots were probed with polyclonal RUNX1 antibody, strip and re-probed with mouse monoclonal GAPDH antibody. C) HeLa cells were co-transfected with P1-Luc and increasing concentrations of RUNX1 expression plasmid using nanojuice transfection reagent. Cells were harvested 18h later for luciferase activity. Normalized data pooled from four independent experiments are presented in bar graph. Asterisks indicate statistically significant increase in promoter activity in response to RUNX1 overexpression as determined by student t-test (*p<0.1, p**<0.01). D) HeLa cells were co-transfected with 200ng of P1-Luc and increasing concentrations of RUNX1 expression vectors. Cells were processed for western blot analysis 18h later and a representative blot is shown.

We next examined direct regulation of RUNX1 promoter by RUNX1 protein using co-transfection experiments in HeLa cells. We and others have reported that HeLa cells do not express endogenous RUNX transcription factors and thus serve as a cell system with zero background. Our results show that wild type RUNX1 P1+5’UTR-Luc responds only modestly to increasing dosage of RUNX1 protein. In contrast, P1+5’UTR-Luc with mutated RUNX sites show a dose dependent 2-5-fold increase in promoter activity (Fig 3A). We further established that strong transcriptional response of mutant promoter is not due to any difference in amount of overexpressed RUNX1 protein. Western blot analysis from parallel plates showed comparable levels of RUNX1 protein in transfected cells (Fig 3B). These data suggest that RUNX motifs in the UTR and the promoter region respond differently to RUNX1 overexpression. To confirm a positive response by RUNX motifs located in the promoter region, we performed co-expression studies with P1 promoter lacking the UTR region (Fig 3C). Interestingly, the P1 promoter containing two RUNX motifs, showed a similar RUNX1-dose dependent increase in activity (Fig 3C and 3D). Taken together our results demonstrate that RUNX1 autoregulates transcriptional activity of P1 promoter through RUNX motifs.

### RUNX1 directly binds to P1 promoter and regulates RUNX1 gene transcription

**Fig 4:**
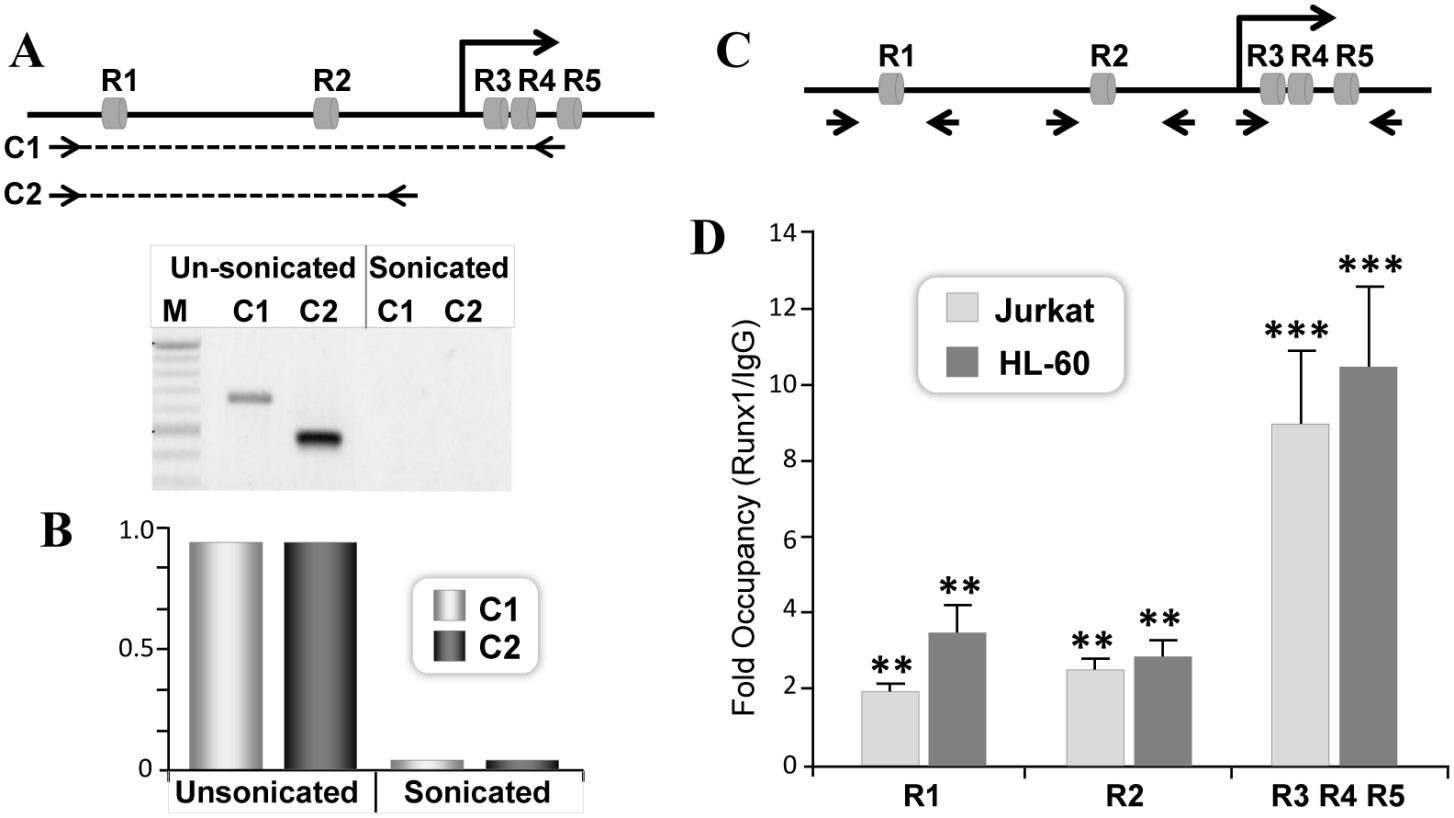
Endogenous RUNX1 protein is recruited to RUNX motifs located in the P1 promoter and the 5’UTR. A) Efficiency of chromatin shearing of the RUNX1 locus in Jurkat cells. Control (unsonicated) and sheared (sonicated) chromatin was subjected to PCR using either (A) traditional or (B) real time qPCR analysis. Primer pair amplifying RUNX1 chromatin loci encompassing R1-R4 (C1) or R1-R2 (C2) is indicated with dashed lines. The amplified 650bp C1 and 500bp C2 region of the RUNX1 gene were detected in control but not in sonicated chromatin. M denote 100bp ladder. B) Lack of intact DNA between the promoter and the UTR RUNX sites in sonicated chromatin was confirmed by real time qPCR. C) Arrowhead indicates relative position of primer pairs used to assess RUNX1 occupancy on promoter and UTR RUNX motifs D) Equivalent amount of chromatin (3U OD260) from Jurkat or HL-60 cells were used for ChIP assays. Chromatin was immunoprecipitated with 2μg of IgG or RUNX1 polyclonal antibody. Site specific occupancy was determined by real time PCR. Mean values of IgG or RUNX1 immunoprecipitated DNA from three independent experiments with three replicates were obtained for each site. Data is expressed as fold changes over IgG in respective cells. Asterisks indicate statistically significant enrichment of DNA immunoprecipitated with RUNX1 antibody (**p<0.01, ***p<0.001).

To investigate whether endogenous RUNX1 is recruited to the P1 promoter, we performed ChIP experiments in hematopoietic cells, in which RUNX1 protein is highly expressed. Chromatin immunoprecipitated from Jurkat cells with RUNX1 antibody was quantified by qPCR. The two RUNX motifs in the P1 promoter (R1 and R2) are separated by 338bp. To evaluate independent occupancy of RUNX1 at each site, we initially optimized our sonication conditions (Fig 4A). Traditional PCR showed successful amplification of P1 promoter fragment encompassing R1 and R2 motifs from unsonicated DNA template but not from sonicated DNA (Fig 4A, lower panel). The effective separation of R1 and R2 motifs in the RUNX1 chromatin was further confirmed by lack of any DNA amplification and detection in real time PCR (Fig 4B). Together these data demonstrate that our experimental conditions allow assessment of independent occupancy of RUNX1 on R1 and R2 motifs.

We find RUNX1 protein is recruited to both R1 and R2 motifs and exhibit significant enrichment over IgG ranging from two to three fold in Jurkat cells (Fig 4D). Binding of RUNX1 protein to these motifs was further confirmed in HL-60 cells (Fig 4D). We also determined RUNX1 protein recruitment at the three RUNX sites located in 5’UTR. Due to their close proximity, we could not assess their occupancy individually (Fig 4C). ChIP assay revealed RUNX1 occupancy of these sites in both Jurkat and HL-60 cells. The total enrichment of the UTR-RUNX sites (R3,R4,R5) is significantly higher relative to R1 and R2 sites (Fig 4D). However, if adjusted per RUNX motif they are equivalent to those of RUNX sites within the promoter (Fig 4D). Occupancy of both promoter and UTR sites is consistent with involvement of these sites in transcriptional regulation shown in Figs 1 and 2.

**Fig 5:**
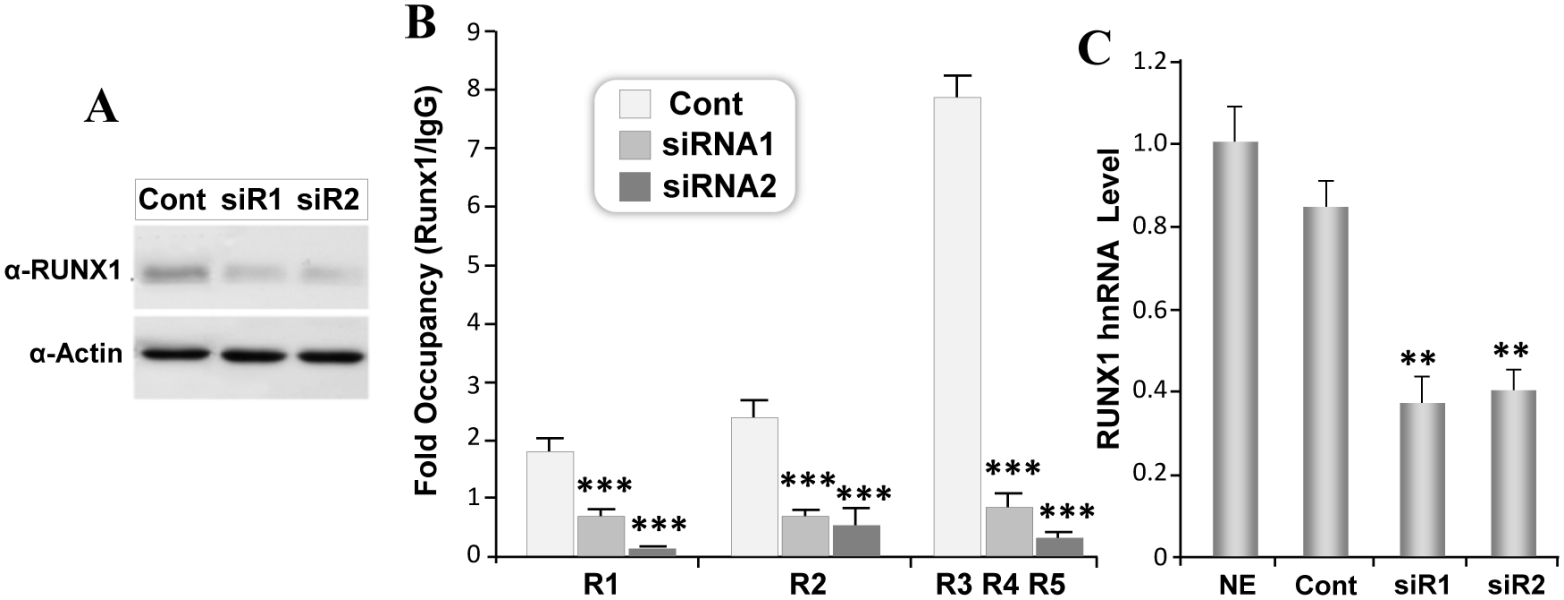
Depletion of endogenous RUNX1 protein in hematopoietic cells decreases occupancy and activity of RUNX1 P1 promoter. A) Jurkat cells were electroporated with 80nM of either control siRNA (Cont) or two different siRNA directed against RUNX1 (siR1 and siR2). Cells were harvested 48h later and equal amount of proteins were resolved by SDS PAGE. Blots were probed with polyclonal antibody against RUNX1, stripped and re-probed with polyclonal antibody against Actin. A representative image of chemiluminescence signal is shown. B) Jurkat cells were electroporated with indicated siRNA for 48h and processed for chromatin immunoprecipitation. Equal amount of immunoprecipitated DNA was subjected to real time PCR amplification. Data was averaged from three independent experiments and is presented as fold over IgG for respective RUNX motifs. C) Jurkat cells electroporated with control or RUNX1 siRNA were pelleted 48h later to isolate RNA. Level of P1 promoter derived RUNX1 hnRNA was determined by qPCR using primer pair located in exon 1 and intron 1. Pooled data from three independent experiments with three replicate each is shown for non electroporeted (NE) and siRNA electroporated Jurkat cells. Asterisks indicate statistically significant decrease in expression of RUNX1 hnRNA (**p<0.01, ***p<0.001).

To determine transcriptional function of endogenous RUNX1 protein, we depleted RUNX1 protein from Jurkat cells. Several small interfering RNA targeting various regions of RUNX1 RNA were initially screened (data not shown). Two siRNA targeting different coding regions of the RUNX1 mRNA showed consistent knock down of RUNX1 protein by 54% and 56% respectively (Fig 5A). ChIP analysis from siRNA treated cells showed a decreased RUNX1 occupancy on all RUNX sites (Fig 5B). However the effect was more pronounced in cells treated with RUNX1 siRNA2 (Fig 5B). To understand consequences of decreased RUNX1 occupancy at these sites we determined RUNX1 gene transcription by evaluating the levels of the RUNX1 heterogeneous nuclear RNA (hnRNA) present in the nucleus. The hnRNA reflects the primary transcript and is normally not degraded by siRNA treatment (Elferink & Reiners, 1996; Köhler & Roos, 2008). We find a significant decrease in P1 promoter derived RUNX1-hnRNA from siRNA treated cells (Fig 5C). Decrease in RUNX1 primary transcript in hematopoietic cells is consistent with the promoter reporter data of a positive autoregulatory loop. Taken together these results indicate direct occupancy of RUNX1 is an integral component of RUNX1 mediated regulation of the P1 promoter.

### SCL transcription factor activates RUNX1-P1 promoter

**Fig 6:**
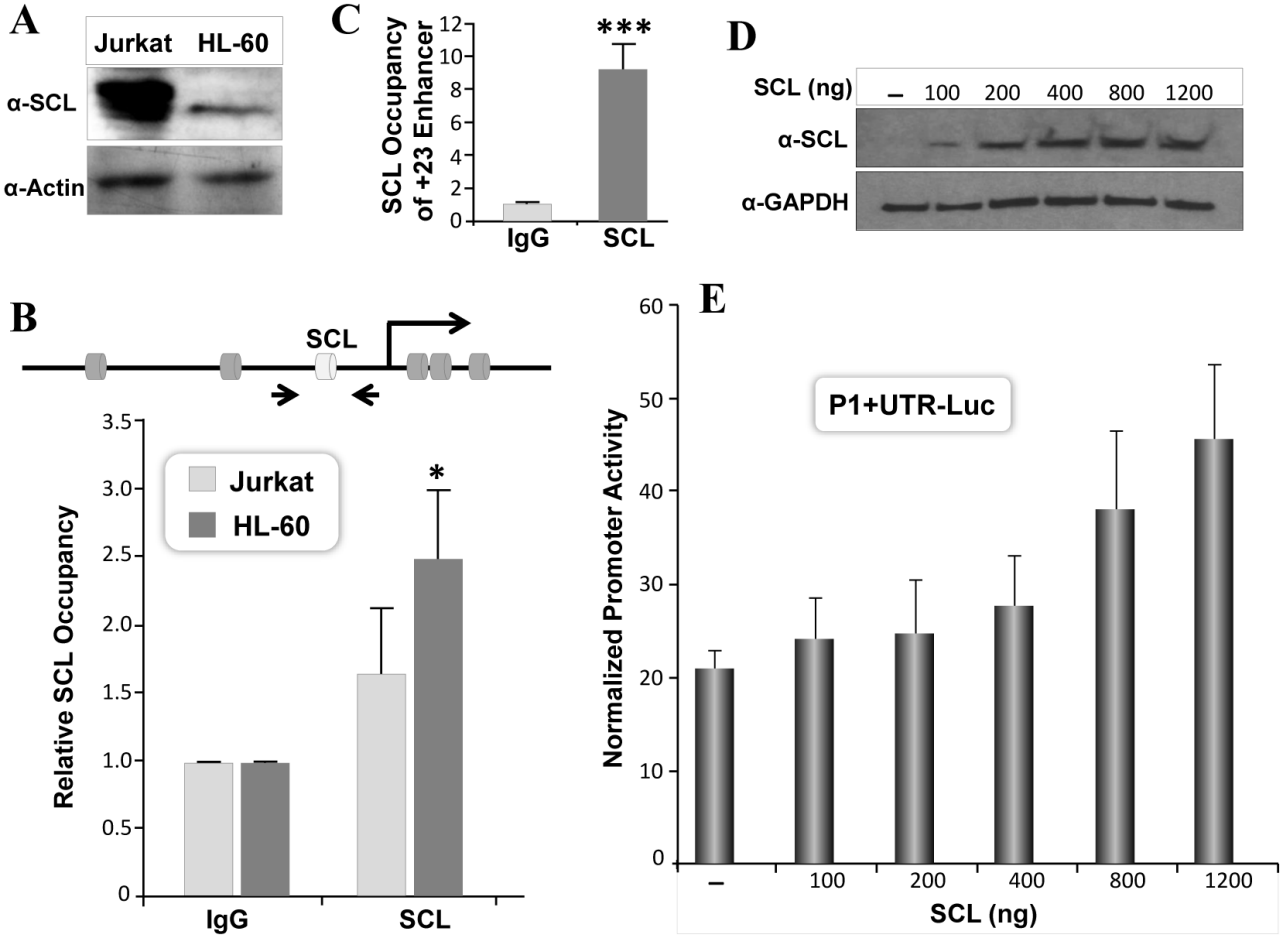
SCL transcription factor activates RUNX1-P1 promoter. A) Equal amount of total protein from Jurkat and HL-60 cells were resolved by SDS-PAGE. Blots were probed with polyclonal SCL antibody, stripped and reprobed for tubulin antigen, used as a loading control. B) Jurkat and HL-60 cells were processed for chromatin immunoprecipitation. SCL occupancy of SCL motif in the RUNX1 P1 promoter was determined by qPCR. The positions of the SCL motif and the primer pair for ChIP assays are indicated in the diagram. Average data from three independent ChIP experiments, with three replicate each is shown as relative to IgG. C) Jurkat cells were processed for ChIP assays exactly as described in (B). Occupancy of SCL motif present in +23 RUNX1 enhancer was determined by qPCR. Average data from three independent ChIP experiments, with three replicate each is shown as relative to IgG. Statistical significance of SCL occupancy is indicated by asterisks (***p<0.001). D) HeLa cells were transiently co-transfected with RUNX1 promoter and indicated amounts of SCL expression vector. Cells were harvested 18h later for western blot analysis. SCL protein was detected by probing blots with SCL antibody. GAPDH is shown as internal loading control in the same blots. E) HeLa cells were co-transfected with 200ng of the RUNX1 P1+UTR Luc and increasing concentration of SCL expression vector. Luciferase activity was determined 18h later and normalized with Renilla luciferase. Bars in graph represent mean values of three independent experiments each performed in triplicate.

Several transcription factors are critical for development of hematopoietic cells. Here we assessed involvement of basic helix-loop-helix containing protein SCL that binds E box motif in the target DNA. The initial two hundred base pairs of the RUNX1-P1 promoter contain an evolutionary conserved E-box motif (Fig 1A). To assess SCL occupancy, ChIP assays were performed in hematopoietic cells. We find SCL is expressed in both Jurkat and HL-60 cells (Fig 6A). However, level of endogenous SCL protein is ten times higher in Jurkat cells. SCL occupancy of the RUNX1-P1 promoter was modest in Jurkat cells (Fig 6B). To assess if this is due to a poor efficiency of immunoprecipitation, we also evaluated SCL occupancy at the +23 RUNX1 enhancer region. We find a 9-fold enrichment of the RUNX1 enhancer region in immunoprecipitated DNA (Fig 6C). Our data is consistent with previous report showing SCL occupancy at the +23 enhancer in Jurkat cells [31]. Thus, the SCL motif in the RUNX1 promoter exhibit a poor occupancy compared to the enhancer region. Interestingly occupancy of SCL on the RUNX1-P1 promoter was significantly higher in HL-60 compared to Jurkat cells (Fig 6B).

These results are consistent with increased basal activity of the RUNX1 P1+UTR promoter in HL-60 cells (Fig 1D and 1E). The ability of SCL to regulate RUNX1 P1+UTR promoter was studied in HeLa cells that lack endogenous expression of SCL protein (Fig 6D). SCL overexpression resulted in a 2-fold increased activity of the RUNX1 P1+UTR promoter (Fig 6E).

**Fig 7:**
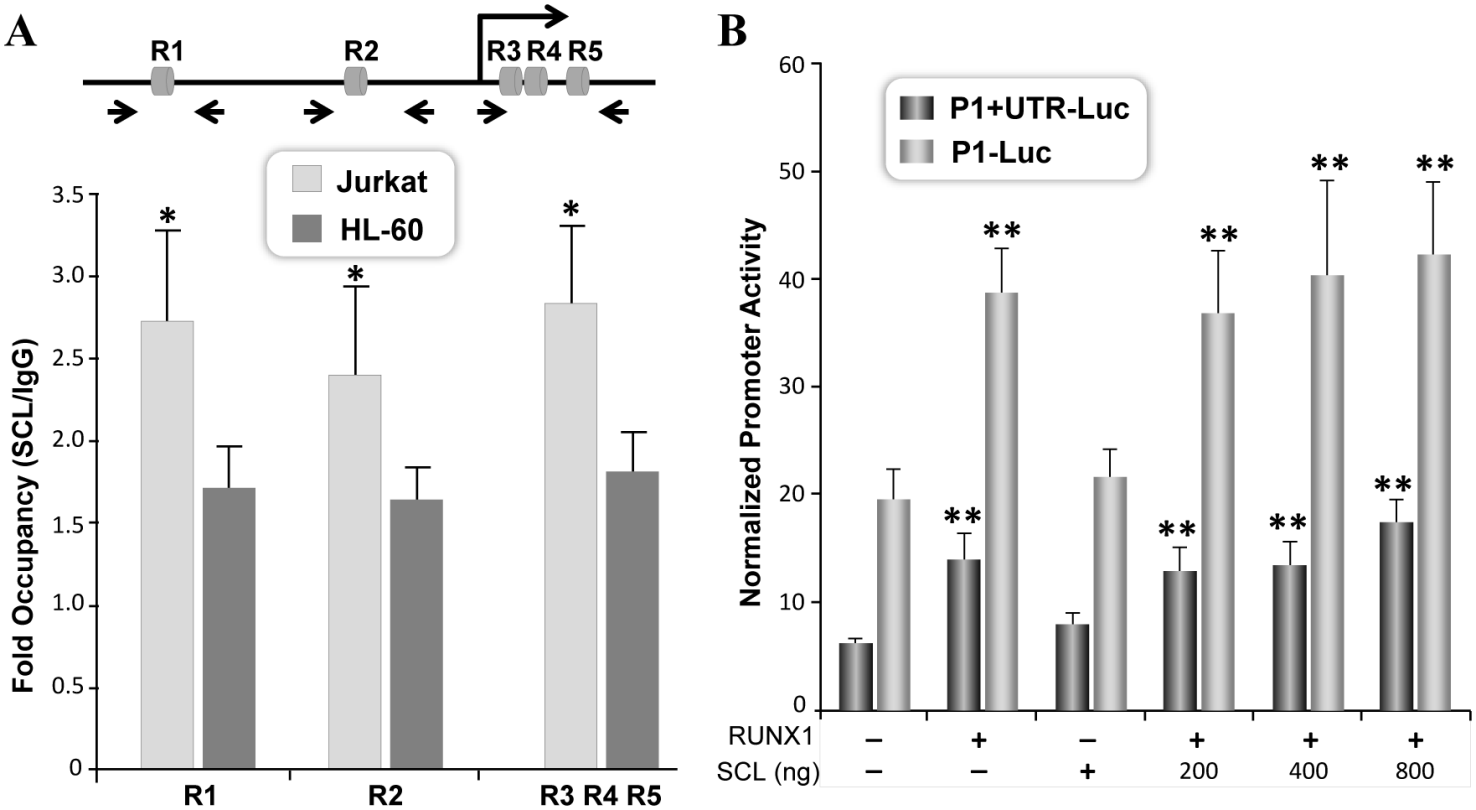
SCL is recruited to regions containing RUNX motifs in the promoter and the UTR. A) Chromatin from Jurkat and HL-60 cells were immunoprecipitated with SCL antibody. DNA regions containing indicated RUNX motifs were amplified by qPCR. Mean values of SCL immunoprecipitated DNA from three independent experiments and three replicates were obtained for each site. Data is presented as fold changes over IgG in Jurkat and HL-60 cells. Statistically significant occupancy of SCL in promoter region containing RUNX motifs was observed only in Jurkat cells (*p<0.1) B) HeLa cells were cotransfected with either P1+UTR-Luc or P1-Luc and 200ng of RUNX1 and SCL expression vectors. For co-expression 200ng of RUNX1 was transfected with increasing amounts of SCL expression vector. Luciferase activity was determined 18h later and values normalized with Renilla luciferase used as internal control. Normalized data pooled from three independent experiments each performed in triplicate, is presented with standard error of the mean. Statistically significant changes in RUNX1 promoter activity is indicated by asterisks (**p<0.01).

Like several hematopoietic transcription factors, SCL forms a molecular complex with RUNX1 protein. Therefore, we assessed interplay between RUNX1 and SCL transcription factor for regulation of the RUNX1-P1 promoter. Interestingly, chromatin immunoprecipitation revealed that SCL protein is recruited to both the P1 promoter and the UTR region that do not contain SCL motif (Fig 7A). These data suggest that SCL is recruited to the RUNX1-P1 promoter by protein-DNA, and protein-protein interaction. We next assessed if RUNX1 and SCL can functionally interact to regulate RUNX1 promoter activity. HeLa cells transfected with RUNX1 or SCL showed activation of both the P1+UTR and P1 promoter (Fig 7B). To our surprise, RUNX1-P1 promoter activity did not change when RUNX1 and SCL were co-expressed (Fig 7B). Thus, co-expression of RUNX1 and SCL does not enhance transcriptional function of RUNX1 protein. In summary, our data show that RUNX1 and SCL proteins can regulate P1 promoter derived transcription of the RUNX1 gene.

## Discussion

In hematopoietic cells two principal isoforms of RUNX1 mRNA are derived from P1 and P2 promoters respectively [26, 43, 44]. In this paper we report that RUNX1 protein regulate transcription of P1-derived RUNX1 mRNA. We find that in hematopoietic cells endogenous RUNX1 protein is bound to both the P1 promoter and the 5’UTR. RUNX motifs present in the proximal promoter activate RUNX1 gene transcription. In contrast, RUNX sites located in the 5’UTR inhibit transcription of a RUNX1-P1 promoter-reporter plasmid. We also show that SCL transcription factor is recruited to RUNX1-P1 promoter and enhances activity of P1 promoter. Taken together, our results demonstrate that in hematopoietic cells RUNX1 gene expression is regulated by a positive feedback mechanism.

The P1 and P2 promoters of the RUNX1 gene in both human and mice are separated by 160kb sequences. Earlier reports have established that a 531bp enhancer region is present in the intron 1 of the mouse RUNX1 gene. This enhancer region located 23.5kb after the RUNX1 translational start site, is highly conserved among different mammalian species. This RUNX1 enhancer can drive hematopoietic cell specific expression of a reporter gene *in vivo* [45]. To define contribution of the promoter sequences in transcription of the RUNX1 gene, we studied sequences of both the P1 promoter and the exon 1 of the RUNX1 gene. We find that a 0.6kb fragment of the P1 promoter and 0.6kb region of exon 1 exhibit a 20-fold higher basal activity in hematopoietic cells when compared with non-hematopoietic cells.

Interestingly, UTR of the RUNX1 gene contains potential repressor regions, as removal of these sequences enhances basal transcription of the RUNX1-P1 promoter. To identify factors contributing to repressor response, we performed an *in silico* analysis of UTR sequences. The UTR region contains three RUNX sites in close proximity. These sites are evolutionary conserved, suggesting a role in the transcriptional regulation of RUNX1 gene. Indeed, chromatin immunoprecipitation revealed that native RUNX1 protein in hematopoietic cells is recruited to these sites. Deletion or mutation of these RUNX motifs results in a 4 to 20-fold increase in activity of the RUNX1-P1 promoter. These data indicates that repressor activity of UTR sequences is due to recruitment of RUNX1 protein to the Runx sites. Interestingly, two evolutionary conserved RUNX motifs are also present within initial 570 bp of the P1 promoter. In hematopoietic cells, endogenous RUNX1 protein is directly recruited to these regulatory motifs. In contrast occupancy of the Runx motifs in the promoter by RUNX1 protein enhances transcriptional activity of the P1 promoter. The ability of RUNX1 protein to serve as transcriptional activator was further confirmed by overexpression and knockdown approaches. Depletion of RUNX1 protein by siRNA in hematopoietic cells, significantly reduces RUNX1 occupancy at these sites, as well as transcription of the RUNX1 gene. Thus, a positive feedback loop is required for expression of RUNX1 gene in hematopoietic cells.

Our over-expression results indicate that RUNX protein activates P1 promoter. However, RUNX motifs located in the promoter and UTR showed a differential response in the presence of RUNX1 protein. The two RUNX sites located in the P1 promoter showed an enhanced transcriptional response to RUNX1 protein. In sharp contrast, the three RUNX motifs located in UTR inhibits transcriptional activation by the RUNX1 protein. These findings are consistent with earlier reports demonstrating that based on promoter and cell context, RUNX proteins can act as either transcriptional activators or transcriptional repressors [46]. Most likely, this differential response by RUNX regulatory motifs is necessary to maintain tight levels of RUNX1 mRNA within hematopoietic tissues. The ability of RUNX proteins to form cell specific and combinatorial regulatory complexes allows either activation or repression of gene transcription [47–50]. For example, RUNX1 protein establishes activating or repressing complexes by physical interaction with other hematopoietic transcription factors such as GATA, SCL, C/EBP, SMAD coactivators and Groucho/TLE, mSin3A and NCoR corepressors [51–56]. Additional parameters, such as chromatin structure and interaction with chromatin modifying proteins also determines the functional response of RUNX1 protein [54]. RUNX1 binding to P1 promoter and the exon 1 region could permit the formation of a chromatin modifying regulatory hub in hematopoietic cells. In fact, previous reports have shown that chromatin looping can bring together both promoter and enhancer regions and that RUNX1 binding to its cognate motif is the initial event in chromosomal looping [45, 57]. Thus, RUNX1 gene transcription is controlled by differential response of RUNX motifs and formation of regulatory complexes with other transcription factors and chromatin modifying proteins.

Several transcription factors including members of the SMAD family, GATA1 and PU.1 regulates RUNX1 gene expression in hematopoietic cells. During hematopoiesis, these transcription factors work cooperatively by either forming a physical and/or functional interaction with RUNX1 protein. In this paper we also studied involvement of SCL transcription factor in regulating P1 promoter derived RUNX1 gene transcription. A putative SCL binding motif is present in the RUNX1 P1 promoter. This SCL motif is located between RUNX sites present in the UTR and the P1 promoter. In hematopoietic cells the endogenous SCL protein is recruited to this site. However, SCL occupancy in the promoter site is rather modest when compared with the SCL motif located in the +23 enhancer of RUNX1 gene. Overexpression of SCL protein in non-hematopoietic cells enhances transcriptional activity of the RUNX1-P1 promoter. Interestingly, SCL protein is recruited to both the UTR and the P1 promoter regions. Our findings are consistent with previous reports that showed physical interaction between SCL and RUNX1 protein [53]. However, co expression of SCL and RUNX1 did not cause synergistic activation of the RUNX1-P1 promoter. Thus, occupancy of SCL at the RUNX1-P1 promoter and enhancer regions may support chromatin conformation that is conducive for RUNX1 gene transcription [45].

In summary, our findings demonstrate that in hematopoietic cells RUNX1 protein is recruited to its own promoter to regulate RUNX1 gene transcription in a positive feedback loop. This auto-regulation is also noted for other members of the RUNX gene family [58, 59]. Thus, auto-regulatory process maybe a key mechanism for sensing and maintaining RUNX1 protein threshold necessary for specification and differentiation of hematopoietic cells.
